# Comparative Evaluation of Arabin Pessary and Cervical Cerclage for the Prevention of Preterm Labor in Asymptomatic Women with High Risk Factors

**DOI:** 10.3390/ijerph15040791

**Published:** 2018-04-18

**Authors:** Panagiotis Tsikouras, George Anastasopoulos, Vasileios Maroulis, Anastasia Bothou, Anna Chalkidou, Dorelia Deuteraiou, Xanthoula Anthoulaki, Georgios Tsatsaris, Arzou Halil Bourazan, George Iatrakis, Stefanos Zervoudis, Georgios Galazios, Lola-Katerina Inagamova, Roland Csorba, Alexander-Tobias Teichmann

**Affiliations:** 1 Department of Obstetrics and Gynecology, Democritus University of Thrace, 68100 Alexandroupolis, Greece; gbmaroul@yahoo.com (V.M.); annachalkidou@yahoo.gr (A.C.); Dr.dorelia@hotmail.com (D.D.); xanthi_vatsidou@hotmail.com (X.A); arzouhalil@hotmail.com (A.H.B.); ggalaz@med.duth.gr (G.G.); lola-katerina@hotmail.gr (L.-K.I.); 2 Medical Informatics Laboratory, Medical School, Democritus University of Thrace, 68100 Alexandroupolis, Greece; anast@med.duth.gr (G.A.); tsatsarisg3@gmail.com (G.T.); 3 Department of Obstetrics and Gynecology, Rea Hospital, 17564 Athens, Greece; natashabothou@windowslive.com (A.B.); szervoud@otenet.gr (S.Z.); 4 Department of Obstetrics and Gynecology, Technological Educational Institute, 17564 Athens, Greece; giatrakis@teiath.gr; 5 Department of Obstetrics and Gynecology, Clinicum Aschaffenburg, Teaching Hospital of University, 97070 Würzburg, Germany; drcsorbaroland@gmail.com (R.C.); buero.teichmann@gmx.de (A.-T.T.)

**Keywords:** cervical Arabin pessary, cerclage, second trimester of pregnancy

## Abstract

Objective: Preterm labor is one of the most significant obstetric problems associated with high rate of actual and long-term perinatal complications. Despite the creation of scoring systems, uterine activity monitoring, cervical ultrasound and several biochemical markers, the prediction and prevention of preterm labor is still a matter of concern. The aim of this study was to examine cervical findings for the prediction and the comparative use of Arabin pessary or cerclage for the prevention of preterm birth in asymptomatic women with high risk factors for preterm labor. Material and methods: The study group was composed of singleton pregnancies (spontaneously conceived) with high risk factors for preterm labor. Cervical length, dilatation of the internal cervical os and funneling, were estimated with transvaginal ultrasound during the first and the second trimesters of pregnancy. Results: Cervical funneling, during the second trimester of pregnancy, was the most significant factor for the prediction of preterm labor. The use of Arabin cervical pessary was found to be more effective than cerclage in the prolongation of pregnancy. Conclusion: In women at risk for preterm labor, the detection of cervical funneling in the second trimester of pregnancy may help to predict preterm labor and to apply the appropriate treatment for its prevention. Although the use of cervical pessary was found to be more effective than cerclage, more studies are needed to classify the effectiveness of different methods for such prevention.

## 1. Introduction

Premature birth is defined as birth between 23 weeks + 5 days and 37 weeks of gestation, occurs in 10–12% of deliveries and is the leading cause of perinatal morbidity and mortality [1,2,3]. When it occurs, it leads to 75% perinatal mortality and morbidity and, in 50%, of cases there are long-term permanent neurological complications [4]. According to Chang’s report, approximately 1.1 million neonates die from preterm birth complications [5]. In 2010, it was estimated that 14.9 million neonates were born in 11.1% of all births globally, 5% in Europe and 18% in Africa [5]. More than 60% of preterm births are observed in countries of South Asia and Sub-Saharan Africa [6]. Finnström suggested that the prolongation of pregnancy especially, to 23–26 weeks of gestation increases the survival rate of premature neonate by 3% [7].

Preterm birth is a clinical syndrome with various causes which can be genetic, maternal (smoking, multiple pregnancies, diabetes mellitus, hypertension cervical insufficiency, and age of <18 or >40), fetal (malpresentation), hormonal, social and environmental and it may be difficult to recognize the exact mechanism that provokes the labor. Until now, it is believed that the majority of preterm births have an idiopathic cause [8,9]. As medicine evolved in the detection of infections, both asymptomatic and symptomatic, the ability to study microorganisms (bacteria, viruses, and parasites) and their action (toxins, immune response, prostaglandins, proteases, etc.) helped to understand various things about the importance of infections in the provocation of preterm birth [8,9]. Although a lot of research has been performed on this important condition, the frequency has increased annually in the last years [6]. Ultrasound examination of the cervix can recognize women with increased risk of preterm birth based on sonographic measurement of the cervical length and funneling of the cervical internal os in the mid trimester and, consequently, prevention and intervention lead to decreased incidence of preterm birth. However, the diagnosis of cervical insufficiency (short cervical length and dilation of internal cervical os) is difficult and there are no existing objective diagnostic criteria [10,11,12]. In cases of cervical insufficiency, the value of cervical cerclage and cervical pessaries is still a matter of controversy [13,14].

The aim of this study was to examine cervical findings for the prediction and the comparative use of Arabin pessary or cerclage for the prevention of preterm birth in asymptomatic women with high risk factors for preterm labor.

## 2. Materials and Methods

In the interval between 2007 and 2012 in a retrospective cohort research study, we studied the cervical length and the dilatation of the internal os of pregnant women in the 1st trimester (10–14 weeks of gestation) and in the 2nd trimester (14–28 weeks of gestation). Ninety-five percent of all women were followed in Teaching Hospital Aschaffenburg of Germany and the rest in the Democritus University, Department of Obstetrics and Gynecology. The study was approved by the scientific committee as a clinical audit in the two departments.

All studied pregnancies were singleton asymptomatic pregnancies with high risk factors in the past such as recurrent miscarriage, previous preterm birth, recurrent vaginal bleeding, cervical surgical procedures, and infections.

Exclusion criteria included: stillbirths, fetal congenital anomalies, uterine anomalies, preeclampsia and metabolic diseases.

The transvaginal measurements were performed by experienced clinicians in the two departments according to standard recommended techniques.

Cervical length less of 2.7 cm, dilatation of the internal os more than 10 mm or indentation V or U shaped of the internal os were determined early as important signs for a cervical insufficiency.

In our pregnant women, we collected the following data: maternal age, parity, past obstetric and gynecological history, cervical length, funneling, cerclage vs. pessary insertion or conservative supervision of pregnancy gestation age at delivery and labor modus.

All study participants had normal findings in the sonographical measurements in the first trimester and were asymptomatic. Women with previous history of abnormal cervical factors were found to have abnormal measurements in reevaluation during the second trimester, especially during 14–28 weeks of gestation. The treatment of the cervical insufficiency was performed either with cerclage or Arabin pessary. In some cases, the performance of both methods was obligatory while in the rest neither was necessary except conservative medical therapy.

This study evaluates the possible role of cervical length, dilatation of the internal cervical os, funneling and the use of cerclage or pessary in avoiding this problem.

Our target was to study the correlation between the mentioned three abnormal parameters and preterm labor under 33 weeks. We defined “early preterm labor” as delivery under 33 weeks, since our patients have a medical history of high-risk factors and all of them had a preterm delivery. We used linear and logistic regression to make statistical analysis. We also tried to find which of the two invasive procedures (pessary and the placement of cervical sutures) is more effective in prolonging the week of delivery and which is less harmful. A *p* value <0.05 was considered as statistically significant.

## 3. Results

In total, 166 women were examined sonographically and we found that 95 (57.2%) had cervical length less than 2.7 cm. Dilatation of the internal cervical os >10 mm was found in 138 (83.1%) women and funneling was found in 51 (30.7%). Thirty-nine women (23.5%) who had transvaginal sonographic assessment of the cervix had preterm labor under 33 weeks. Only 20 of 166 women of the study (12%) had a cervical cerclage procedure and 124 of them (74%) had a pessary placement. About the mode of delivery, 61 participants (36.7%) had normal vaginal delivery, 9 (5.4%) had vacuum extraction and 96 (57.8%) had a low segment cesarean section (Table 1).

**Figure 1:**
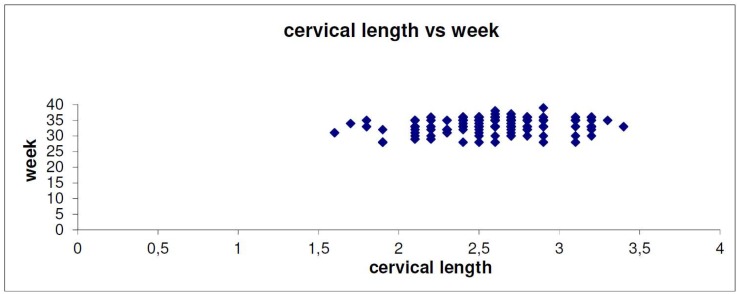
The association of cervical length and pregnancy week (*p* = 0.534).

**Figure 2:**
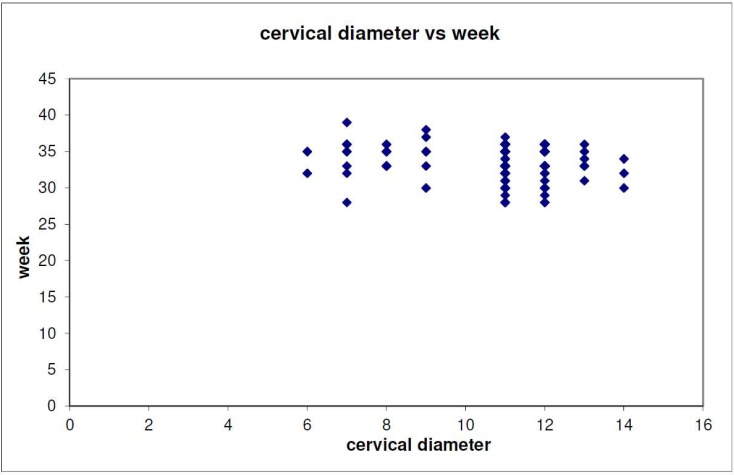
The association of cervical diameter and pregnancy week (*p* = 0.210).

Table 2 describes the relationship between early labor under 33 weeks and the results of the transvaginal sonography. Logistic regression was used for the need of the specific study. As shown in Table 2, the prediction of labor under 33 weeks of gestation cannot be based on the results of transvaginal ultrasonography about cervical length and dilatation of the internal cervical os, since there is not enough statistical significance to support these specific cases. Of the 39 women who had an early preterm delivery, 25.6% had abnormal cervical length, whereas 21.12% had a normal one. However, this inference cannot be considered as a factor of early preterm delivery (*p* = 0.534 > 0.05). Women who had dilatation of cervical os >10 mm (25.3%) were more likely to have preterm delivery than those who did not (14.2%). However, for the same reason, a *p* value of 0.210 means that this factor cannot be considered. These results can also be perfectly explained in Figure 1 and Figure 2. Concluding, if a woman has cervical diameter >10 mm or cervical length <2.7 cm or both, we cannot be definitely sure that she will have a delivery under 33 weeks. Funneling seems to be a predicting factor of great importance. It was found that there is a relationship between funneling and delivery under 33 weeks (YES = 39.2%, NO = 19.8%, *p* < 0.05). The treatment modus (cervical cerclage and pessary) were also checked in Table 3. Fifty percent of the ten women who had a placement of a cervical suture delivered under 33 weeks, whereas only 19.6% those who did not have a cerclage procedure had preterm labor under 33 weeks. The *p* value is <0.05 and this means that the result is statistically significant. In addition, it is concluded that the incidence of early preterm labor was significantly higher in women with cervical cerclage compared to women with no medical intervention. A group of women had a pessary placement. The outcome from the specific group was very encouraging. It was found that pessary does not affect the week of delivery in an adverse way. According to our findings, statistical significance of age (*p* = 0.419), parity (*p* = 0.295) and labor modus was not confirmed.

As presented in Table 4, if a funneling exists in a woman, there is a three times higher risk of early preterm (Odds Ratio (OR) = 3.260, with Confidence Interval (CI): 1.544–6.881 and *p* < 0.05) and if she has a cervical cerclage placement, there is a four times higher risk of labor under 33 weeks. The placement of the pessary has a 1.7 higher risk of labor (OR = 1.750, CI: 0.503–2.721 and *p* > 0.05) under 33 weeks and it is not statistically significant, which means that it does not affect negatively as the cerclage procedure.

Finally, in Table 5, the groups of women with one, two or no invasive characteristics are examined. Forty women with both cerclage and pessary had a great possibility of early preterm delivery (*p* < 0.05). For women who had no surgical intervention and had only pessary, there was less possibility (*p* = 0.139) for preterm delivery, which means that pessary is a procedure that does not affect negatively the week of labor. As far as the group of women who underwent a cerclage is concerned, there is no statistical significance (*p* = 0.401), which means that it cannot cause early preterm labor. Unfortunately, there is not enough evidence to rule it out (because there were only two cases).

On the other hand, judging from the second and the last line of Table 4, we can see that pessary placement (second line) does not cause preterm labor under 33 weeks, but what make women of the last group have a delivery under 33 weeks is that they have also a placement of a cervical suture during the second trimester and it is presumed harmful for these women concerning their preterm labor.

By using logistic regression, we also managed to create three equations, in which we can predict if the week of labor is under 33, taking into account only the physical features (Equation (1)), only the invasive characteristics (Equation (2)), or both physical and invasive characteristics. If every feature is substituted by 0 or 1 according to the sample, then multiplied with the specific coefficient and added to the constant number of the first column, we find a number which will be very close to 0 or 1, representing the week of labor (early preterm labor <33 is 0, preterm labor is 1). The formulas are the following:

(1) Week=cervical length×(0.136)+cervical diameter×(−0.242)+funneling×(−1.112)+1.745

(2) Week=pessary×(0.011)+cerclage×(−1.397)+1.387

(3) Week=cervical length×(0.123)+cervical diameter×(−0.398)+funneling×(−0.983)+pessary×(0.503)+cerclage×(−0.740)+1.568

with cervical length (<2.7 = 0, >2.7 = 1), cervical diameter (>10 = 0, <10 = 1), funneling (yes = 1, no = 0), cerclage (yes = 1, no = 0), and pessary (yes = 1, no = 0).

## 4. Discussion

The preterm labor contribution to adverse outcome is largely related to pregnancy age at delivery. Despite continuous research, not a single effective method for satisfactory prognosis of preterm labor and prevention of preterm birth exists. Main risk factors have been suggested to increase the risk of prematurity, however, in most cases, it is not exactly possible to recognize clearly identifiable risk factors. In 25% of cases, the clinical symptomatology does not occur simultaneously with uterine activity [15]. Cervical evaluation by transvaginal ultrasonography in early pregnancy (first and second trimester) is a useful predictor of the risk for spontaneous preterm labor in asymptomatic pregnant women [16].

The cervix is a spindle-shaped structure, around 2 cm long and 1–2 cm wide with main fibrous structure elements, including collagen 80% type I, 20% type III and only a small amount of smooth cells about 10% [17]. Normally, in the late pregnancy prior to early phases of delivery at full term pregnancy, the cervix undergoes cervical ripening depending on biochemical changes, such as reduction of collagen synthesis and increased collagenase activity, which leads to delivery of the fetus [18,19]. In cases of preterm labor the cervical changes like cervical softening associated with painless dilatation, and shortening is explained by increased synthesis of interleukin (IL)-6 amd IL-8 and prostaglandin synthesis, and monocyte chemotactic protein I in absence of infection [20,21]. Asymptomatic pregnant women more commonly in the second than in the first trimester have a prediction to preterm labor in cases with abnormal sonographic cervical findings including the length of cervix (distance between the triangular area of echo density at the external cervical os and the V shaped notch at the internal one) [22,23].

According to previously published literature, the shortened cervical length is mainly a powerful biological marker of preterm labor andg a strong inverse association between cervical length and risk of preterm labor exists [24,25,26]. This risk is especially very high in cases with length less than 15 mm and is equivalent in multiple pregnancies with occurrence at 25 mm [27,28].

Based on our findings, we confirm that, in asymptomatic pregnant women with risk factors mainly in the past, the performance of transvaginal ultrasound cervical assessment in the second trimester is of great importance, even if the ultrasound examination in the first trimester was without abnormal findings. In our cohort, cervical funneling is a main prognostic factor of the prediction of preterm labor: it has three times higher risk compared to the rest of the participants with only abnormal cervical length. This finding is in accordance to previously published papers [29,30].

Concerning the other examinations parameter with cervical length, we found no statistically significant correlation between this one and preterm labor. This finding is surprisingly against previously published literature and our report 10 years ago in which we included abnormal ultrasound cervical assessments in the first and second trimester [31,32,33,34].

The fact that the frequency of preterm birth is not decreasing and is associated with significant costs, the aim of preventing treatment with cervical cerclage, progesterone, and vaginal pessaries is to prolong the duration of pregnancy and to decrease the perinatal morbidity and mortality [35,36,37,38,39].

### Prevention of Preterm Birth

In recent years, the use of vaginal pessary has been returned to the forefront. The vaginal pessary that is used is Arabin pessary [40]. A multifactorial research in Spain proved that, in women with cervical length <25 mm in 18–22 weeks of pregnancy, the use of pessary decreased premature birth in 34 weeks of gestation by 88% and also decreased neonate complications [41]. On the other hand, a new study proved that the preventive usage of vaginal pessary in twin pregnancies does not improve the perinatal result [42]. We have to wait also for the result of other studies to prove or not its usage, and whether the use of vaginal pessaries in women with decreased length of the cervix is helpful. When they do not want or they cannot undergo cervical cerclage, particularly for women with decreased cervical length after 25 weeks of gestation, cervical cerclage is not helpful. We found no negative association between early preterm labor and pessary and so we can confirm the effectiveness of this conservative procedure. No complications were noticed [43,44]. In cases where a cerclage was performed, the contribution of the surgical procedure was not positive to prolong the pregnancy duration, although no serious side effects occurred. However, the necessity of cerclage was not confirmed in our participants, similar to several previously published papers [45,46,47,48].

## 5. Conclusions

Transvaginal sonographic detection of cervical funneling in the second trimester of pregnancy is the most important marker for the prediction of preterm labor. Arabin pessary (probably in combination with progesterone) seems to be effective in its prevention. Further multicenter studies are necessary to confirm these findings and determine as guidelines in the future.

## Figures and Tables

**Table 1:** Cervical characteristics of participants and labor modus.

		# of Women	%
Cervical length <2.7 cm		95	57.2
Dilatation of internal cervical os ≥10 mm		138	83.1
Cervical funneling		51	30.7
Cervical cerclage		20	12
Pessary		124	74
Preterm labor <33 weeks		39	23.5
Mode of delivery	Normal vaginal delivery	61	36.7
	Vacuum extraction	9	5.4
	Cesarean section	96	57.8

**Table 2:** Cervical characteristics of the early preterm labor (<33 weeks) women. Bold type means statistical significance.

Cervical Characteristics		# of Women	%	*p*
Cervical length	<2.7 cm	24	25.6	0.534
	≥2.7 cm	15	21.12	
Dilatation of internal cervical os	>10 mm	35	25.3	0.210
	≤10 mm	4	14.2	
Cervical funneling	No	19	19.8	**<0.001**
	Yes	20	39.2	

**Table 3:** Treatment of cervical insufficiency (cervical cerclage, pessary) of the early preterm labor (<33 weeks) women. Bold type means statistical significance.

Caption		# of Women	%	*p*
Cervical cerclage	No	29	19.6	**0.003**
	Yes	10	50	
Pessary	No	9	21.4	0.717
	Yes	30	24.2	

**Table 4:** Correlation between preterm labor and cervical characteristics expressed as odds ratio (OR) with 95% confidence intervals (CI).

Cervical characteristics	OR	CI	*p*
Cervical length <2.7 cm	0.792	0.38–1.651	0.537
Dilatation of internal cervical os >10 cm	2.039	0.665–6.285	0.215
Funneling	3.260	1.544–6.881	0.002
Cervical cerclage	4.034	1.535–10.603	0.005
Pessary	1.170	0.503–2.721	0.715

**Table 5:** Labor outcome after treatment of cervical insufficiency (cervical cerclage, pessary). Bold type means statistical significance.

Pessary	Cerclage	# of Women	# of Early Preterm Labor Women	*p*
No	No	40	8	**<0.05**
Yes	No	106	21	0.139
No	Yes	2	1	0.401
Yes	Yes	18	9	<0.05
